# Impact of Hydrogel Spacer on Radiation Proctitis After Prostate Cancer Radiation Therapy: A Large-Scale Claims Database Analysis

**DOI:** 10.1016/j.adro.2026.102145

**Published:** 2026-07-17

**Authors:** Kazuya Takeda, Shinsaku Okuda, Rei Umezawa, Takaya Yamamoto, Noriyoshi Takahashi, Ken Takeda, Keiichi Jingu

**Affiliations:** a Department of Radiation Oncology, Tohoku University Graduate School of Medicine, Miyagi, Japan; b Department of Radiation Oncology, South Miyagi Medical Center, Shibata, Miyagi, Japan; c Course of Radiological Technology, Health Sciences, Tohoku University Graduate School of Medicine, Sendai, Miyagi, Japan

## Abstract

**Purpose:**

We evaluated the real-world utility of hydrogel spacer placement for reducing radiation proctitis following prostate cancer radiation therapy using a large-scale claims database.

**Methods and Materials:**

We analyzed male patients from a Japanese administrative claims database (DeSC Database) who initiated primary radiation therapy between July 2018 and December 2024. Propensity score weighting using the average treatment effect on the treated method was applied to balance baseline characteristics between spacer users and nonusers. The primary endpoint was the occurrence of endoscopic gastrointestinal hemostasis, and the secondary endpoint was the diagnosis of radiation proctitis. Fine-Gray models were used to estimate subdistribution hazard ratios (sHRs) with death as a competing risk.

**Results:**

Of 7860 eligible patients, 1754 (22.3%) underwent spacer placement. For the primary endpoint (endoscopic hemostasis), the spacer group showed a statistically significant lower risk (sHR 0.26; 95% CI, 0.10-0.69; *P* = .007). The cumulative incidence at 36 months was 0.8% in the spacer group versus 2.3% in the nonspacer group. For the secondary endpoint (diagnosed proctitis), the sHR was 0.29 (95% CI, 0.16-0.53; *P* < .001), with a 36-month cumulative incidence of 1.4% versus 4.8%, respectively.

**Conclusions:**

This large-scale real-world analysis demonstrates that hydrogel spacer placement is associated with a significantly lower incidence of severe radiation proctitis requiring endoscopic hemostasis.

## Introduction

Radiation therapy is one of the primary curative treatment modalities for localized prostate cancer, alongside surgery. Radiation proctitis is a major adverse event following radiation therapy for prostate cancer, which may require invasive interventions such as endoscopic hemostasis, blood transfusion, or surgery in severe cases.^1^^,^^2^ To address this, hydrogel spacer placement between the prostate and the rectum has been introduced in recent years, with reports indicating a reduction in proctitis.^3^^,^^4^

However, the widespread adoption of image guided radiation therapy, intensity modulated radiation therapy (IMRT), hypofractionation, and particle therapy has lowered rectal radiation doses, suggesting a corresponding decrease in the frequency of severe radiation proctitis.^5^, ^6^, ^7^, ^8^, ^9^, ^10^ Consequently, the efficacy of spacer placement in this modern treatment landscape has not been fully elucidated. Furthermore, existing evidence on hydrogel spacer placement has been limited to single-center reports or clinical trials using single treatment modalities,^3^^,^^4^^,^^11^^,^^12^ limiting the generalizability of the findings.

This study was conducted to evaluate the utility of hydrogel spacer placement in preventing radiation proctitis in a real-world setting by analyzing a large-scale health insurance claims database.

## Methods and Materials

### Ethical approval and consent to participate

This study was an observational study using an anonymized database and was conducted in accordance with the Declaration of Helsinki. The requirement for informed consent from patients was waived. This study was conducted with the approval of the Ethics Committee of the Tohoku University Graduate School of Medicine (approval number: 2025-1-452, approval date: September 24, 2025).

### Data source

This study utilized the DeSC database (DeSC Healthcare), a commercial claims database in Japan. Japan employs a universal health insurance system, requiring all residents to enroll in a public health insurance plan. The DeSC database consists of data provided by multiple insurers, including Society-managed health insurance for corporate employees and their dependents, National Health Insurance for the self-employed and retirees, and the Medical Care System for the Elderly in the Latter Stage of Life for those aged 75 and older. This enables the longitudinal tracking of all insurance-covered medical procedures and drug prescriptions. For this study, the data set for analysis was obtained on September 5, 2025.

### Study population

The date when the confirmed diagnosis of prostate cancer (International Classification of Diseases, 10th Revision [ICD-10] code: C61) was first recorded was defined as the diagnosis date. We excluded patients with an observation period of less than 1 year before the diagnosis. Next, male patients who underwent prostatectomy or radical radiation therapy, defined as external beam radiation therapy with 4 or more fields, stereotactic body radiation therapy (SBRT), particle therapy, or brachytherapy, within 1 year of the diagnosis date, were identified. The earliest of these treatments was defined as the primary treatment. For this reason, patients who underwent radiation therapy following prostatectomy were assigned to the surgery group rather than the radiation therapy group. To limit the study population to localized patients with prostate cancer, we excluded those with diagnoses of other malignancies, lymph node metastases, or distant metastases within 1 year before or during the month of primary treatment. The main analysis targeted cases where primary treatment began between July 2018 and December 2024, corresponding to the period after hydrogel spacer placement was approved for insurance coverage in Japan (June 25, 2018). The final study population for the main analysis consisted of patients who received radical radiation therapy. Patients who underwent surgery were used as a reference group to verify the specificity of the outcomes and to estimate the baseline risk of these outcomes not directly attributable to radiation therapy.

### Exposure and covariates

The primary exposure was the presence or absence of hydrogel spacer placement before radiation therapy. The spacer group was defined as cases where the procedural fee or material costs for hydrogel spacer placement were billed within 90 days before the start of radiation therapy.

All covariates were defined based on information at the start of primary treatment. Patient characteristics included age, treatment year, and insurance type. Insurance types were classified into Society-managed health insurance (mainly for employees of large corporations), National Health Insurance (mainly for the self-employed), and the Medical Care System for the Elderly in the Latter Stage of Life (for those aged 75 and older). Comorbidities and concomitant medications were assessed based on data from the year preceding the primary treatment. Hypertension and diabetes, as common comorbidities associated with vascular status, were defined by the presence of at least 1 confirmed diagnosis code and 1 corresponding medication prescription code. Liver cirrhosis and inflammatory bowel disease, less common conditions that have been linked to a higher risk of rectal adverse events in previous studies,^13^^,^^14^ were defined by at least 1 confirmed diagnosis code. Concomitant medications evaluated included warfarin, direct oral anticoagulants, and antiplatelet agents, defined by the presence of 2 or more corresponding prescriptions.

Treatment-related factors included the facility type and the use of androgen deprivation therapy before radiation therapy. Facilities were classified into Prefectural Designated Cancer Care Hospitals (designated by the Minister of Health, Labour and Welfare as providing advanced cancer care) and other general hospitals. For appropriate classification, the 2 National Cancer Center hospitals in Japan, which are originally classified into an independent category, were also grouped with Prefectural Designated Cancer Care Hospitals in this study. For radiation therapy modalities, a series of treatment sessions with no break of 14 days or more was considered 1 treatment course, and classification was based on the therapies included in that course. In cases involving multiple modalities in a single treatment course, the representative modality was determined based on the following order of priority: brachytherapy, particle therapy, SBRT (ultrahypofractionation), IMRT, and 3-dimensional conformal radiation therapy. Brachytherapy was subclassified as brachytherapy alone or brachytherapy combined with external beam radiation therapy, depending on whether external beam radiation therapy was billed within the course. IMRT was subclassified as conventional fractionation (≥29 treatment days) or moderate hypofractionation (≤28 treatment days).

### Endpoints

The primary endpoint was defined as the first occurrence of endoscopic gastrointestinal hemostasis following the initiation of radiation therapy. This served as a surrogate endpoint for moderate to severe radiation proctitis requiring invasive interventions. Although management strategies for radiation proctitis include topical rectal medications, blood transfusions, or rectal surgery, these are frequently performed for conditions unrelated to radiation therapy, such as hemorrhoids. Therefore, we limited the primary endpoint to endoscopic hemostasis. However, since endoscopic hemostasis may include procedures for gastrointestinal ulcers or lesions unrelated to radiation therapy, the incidence of the same event in the surgery group was also investigated as a reference. For the surgery reference group, both death and any subsequent radiation therapy claim were treated as censoring events to avoid contamination by postoperative radiation therapy, and the results were presented descriptively without baseline adjustment.

The secondary endpoint was the first confirmed diagnosis of radiation proctitis following the initiation of radiation therapy, regardless of whether endoscopic hemostasis was billed. The incidence of this event in the surgery group was similarly assessed for reference.

### Statistical analysis

Patient characteristics were summarized for the hydrogel spacer and nonspacer groups; continuous variables were presented as medians and interquartile ranges, and categorical variables as numbers and percentages. We used the standardized mean difference (SMD) to evaluate the balance of covariates between groups. To evaluate the association between hydrogel spacer use and outcome occurrence, we used inverse probability of treatment weighting (IPTW) to estimate the average treatment effect on the treated. Propensity scores were calculated using a logistic regression model including all the covariates. Under the average treatment effect on the treated approach, each nonspacer patient was assigned an individual weight so that the weighted nonspacer group reproduced the baseline characteristics of the spacer group. Because these weights are continuous, the weighted patient counts are not integers.

The cumulative incidence of events was estimated using the Cumulative Incidence Function, treating death as a competing risk. We used the Fine-Gray subdistribution hazard model for group comparisons to calculate the subdistribution hazard ratio (sHR) and 95% CI. As a sensitivity analysis, crude sHRs without IPTW adjustment were calculated. Additionally, subgroup analysis stratified by baseline characteristics was conducted and presented in forest plots. Subgroups with fewer than 5 events, or with zero events in either group, were excluded from the analysis due to model instability. Statistical significance was defined as a 2-sided *P* value <.05.

Data extraction and processing were performed using Python version 3.9.6 (Python Software Foundation) and DuckDB library version 1.4.1 (DuckDB Labs). All statistical analyses were performed using R version 4.5.1 (R Foundation for Statistical Computing).

## Results

A total of 7860 patients who initiated radical radiation therapy between July 2018 and December 2024 were included in the analysis. Of these, 1754 patients (22.3%) underwent hydrogel spacer placement. Table 1 shows the baseline characteristics of these patients, stratified by hydrogel spacer use. Patients without spacer placement had higher proportions of 3-dimensional conformal radiation therapy and IMRT, whereas those with spacer placement had higher proportions of SBRT, particle therapy, and brachytherapy (SMD: 1.13). Table 2 presents the patient characteristics after inverse probability weighting. After applying weights to the nonspacer group to balance against the 1754 spacer patients, the effective sample size of the weighted nonspacer cohort was 1878.2. Most baseline covariates were well balanced between the groups after weighting; however, only the SMD for the facility type remained above 0.1, at 0.24. We also identified 8583 patients who underwent surgery during the same period as a reference group.

**Table 1:** Patient characteristics before weighting

Variable	No spacer (n = 6106)	Spacer (n = 1754)	SMD
Age, years, median (IQR)	77 (71-80)	76 (70-79)	0.18
Treatment year, n (%)			0.35
2018	316 (5.2)	47 (2.7)	
2019	853 (14.0)	107 (6.1)	
2020	1236 (20.2)	289 (16.5)	
2021	1254 (20.5)	399 (22.7)	
2022	1479 (24.2)	566 (32.3)	
2023	806 (13.2)	287 (16.4)	
2024	162 (2.7)	59 (3.4)	
Health insurance, n (%)			0.14
Employees’ health insurance	261 (4.3)	111 (6.3)	
National health insurance	2227 (36.5)	710 (40.5)	
Medical care system for the elderly	3618 (59.3)	933 (53.2)	
Comorbidities, n (%)			
Hypertension	3849 (63.0)	1065 (60.7)	0.05
Diabetes	1239 (20.3)	289 (16.5)	0.10
Liver cirrhosis	48 (0.8)	17 (1.0)	0.02
Inflammatory bowel disease	14 (0.2)	10 (0.6)	0.05
Medication use, n (%)			
Warfarin	109 (1.8)	17 (1.0)	0.07
DOACs	370 (6.1)	92 (5.2)	0.04
Antiplatelet agents	1379 (22.6)	359 (20.5)	0.05
Prefectural designated cancer hospital, n (%)	478 (7.8)	264 (15.1)	0.23
Neoadjuvant ADT, n (%)	5223 (85.5)	1435 (81.8)	0.10
Treatment modality, n (%)			1.13
3D-CRT	1380 (22.6)	127 (7.2)	
IMRT (conventional fractionation)	1920 (31.4)	143 (8.2)	
IMRT (moderate hypofractionation)	1588 (26.0)	328 (18.7)	
SBRT	126 (2.1)	91 (5.2)	
Particle therapy	855 (14.0)	766 (43.7)	
Brachytherapy alone	207 (3.4)	291 (16.6)	
Brachytherapy with external RT	30 (0.5)	8 (0.5)	

*Abbreviations:* ADT = androgen deprivation therapy; 3D-CRT = 3-dimensional conformal radiation therapy; DOACs = direct oral anticoagulants; IMRT = intensity modulated radiation Therapy; IQR = interquartile range; RT = radiation therapy; SBRT = stereotactic body radiation therapy; SMD = standardized mean difference.

Values are presented as median with IQR for continuous variables and number with percentage for categorical variables.

**Table 2:** Patient characteristics after weighting (ATT)

Variable	No spacer (n = 1878.2)	Spacer (n = 1754)	SMD
Age, years, median (IQR)	76 (70-79)	76 (70-79)	0.02
Treatment year, n (%)			0.07
2018	47.7 (2.5)	47 (2.7)	
2019	108.1 (5.8)	107 (6.1)	
2020	306.8 (16.3)	289 (16.5)	
2021	455.6 (24.3)	399 (22.7)	
2022	565.7 (30.1)	566 (32.3)	
2023	313.4 (16.7)	287 (16.4)	
2024	80.8 (4.3)	59 (3.4)	
Health insurance, n (%)			0.06
Employees’ health insurance	146.6 (7.8)	111 (6.3)	
National health insurance	740.4 (39.4)	710 (40.5)	
Medical care system for the elderly	991.3 (52.8)	933 (53.2)	
Comorbidities, n (%)			
Hypertension	1119.1 (59.6)	1065 (60.7)	0.02
Diabetes	295.5 (15.7)	289 (16.5)	0.02
Liver cirrhosis	18.5 (1.0)	17 (1.0)	0.002
Inflammatory bowel disease	4.6 (0.2)	10 (0.6)	0.05
Medication use, n (%)			
Warfarin	17.6 (0.9)	17 (1.0)	0.003
DOACs	97.5 (5.2)	92 (5.2)	0.002
Antiplatelet agents	382.5 (20.4)	359 (20.5)	0.003
Prefectural designated cancer hospital, n (%)	461.8 (24.6)	264 (15.1)	0.24
Neoadjuvant ADT, n (%)	1550.6 (82.6)	1435 (81.8)	0.02
Treatment modality, n (%)			0.08
3D-CRT	127.6 (6.8)	127 (7.2)	
IMRT (conventional fractionation)	137.8 (7.3)	143 (8.2)	
IMRT (moderate hypofractionation)	312.3 (16.6)	328 (18.7)	
SBRT	87.0 (4.6)	91 (5.2)	
Particle therapy	866.0 (46.1)	766 (43.7)	
Brachytherapy alone	339.5 (18.1)	291 (16.6)	
Brachytherapy with external RT	8.0 (0.4)	8 (0.5)	

*Abbreviations:* ATT *=* average treatment effect on the treated; 3D-CRT = 3-dimensional conformal radiation therapy; ADT = androgen deprivation therapy; DOACs = direct oral anticoagulants; IMRT = intensity modulated radiation therapy; IQR = interquartile range; RT = radiation therapy; SBRT = stereotactic body radiation therapy; SMD = standardized mean difference.

Values are presented as median with IQR for continuous variables and number with percentage for categorical variables.

**Figure 1:**
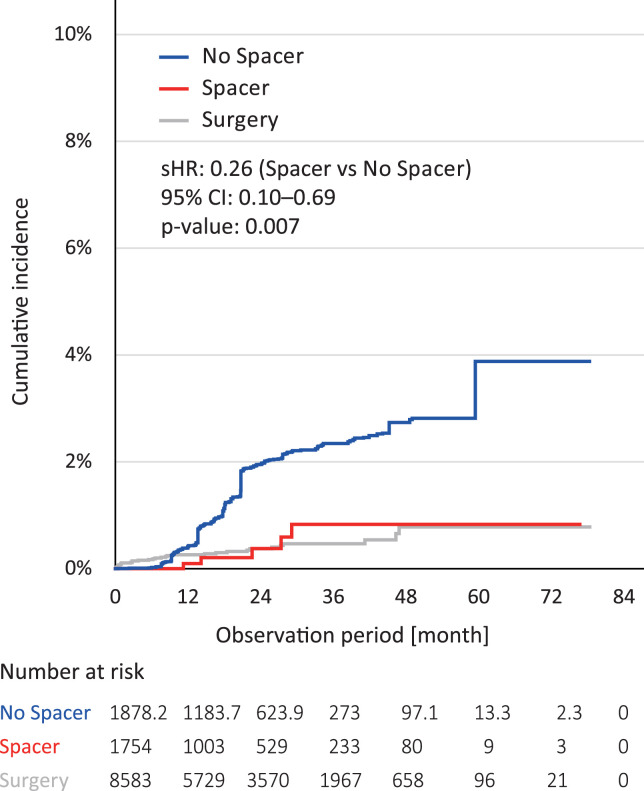
Cumulative incidence of endoscopic gastrointestinal hemostasis in the propensity score-weighted cohort. The cumulative incidence curves after inverse probability of treatment weighting represent the first occurrence of endoscopic gastrointestinal hemostasis after the initiation of radiation therapy. The subdistribution hazard ratio (sHR) and 95% CI were estimated using a Fine-Gray subdistribution hazard model with death as a competing risk.

**Figure 2:**
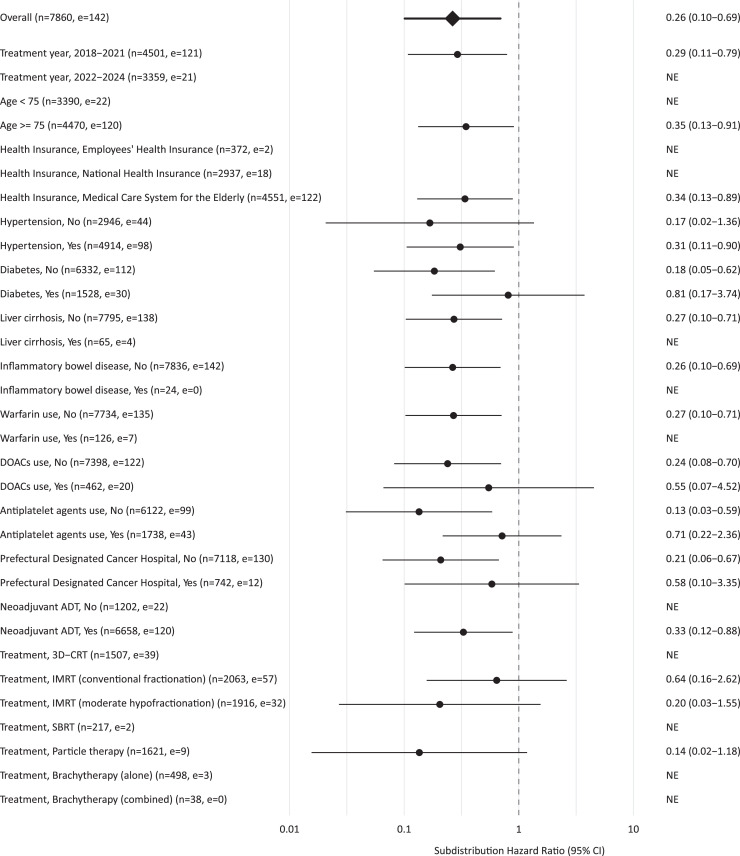
Forest plot of subdistribution hazard ratios for endoscopic gastrointestinal hemostasis across patient subgroups. The subdistribution hazard ratio and 95% CI for the hydrogel spacer group compared with the nonspacer group in each subgroup are shown. “Not Estimable (NE)” indicates subgroups where the hazard ratio was not calculated due to zero events in one or both arms or a total number of events fewer than 5. *Abbreviations:* 3D-CRT = three-dimensional conformal radiation therapy; ADT = androgen deprivation therapy; DOACs = direct oral anticoagulants; e = number of events; IMRT = intensity modulated radiation therapy; n = number of patients (before weighting); SBRT = stereotactic body radiation therapy.

The median follow-up period for the 7860 patients in the radiation therapy cohort was 19.1 months. Figure 1 shows the cumulative incidence of the primary endpoint, endoscopic hemostasis, in the weighted cohort. The sHR for the hydrogel spacer group was 0.26 (95% CI, 0.10-0.69, *P* = .007). The cumulative incidence of events in patients with and without hydrogel spacer placement was 0.2% versus 1.1% at 18 months, and 0.8% versus 2.3% at 36 months, respectively. For comparison, in the reference group of 8583 radical prostatectomy patients, the cumulative incidence of endoscopic hemostasis was 0.3% at 18 months and 0.5% at 36 months. Figure 2 shows a forest plot of sHRs across subgroups. In all 20 evaluable subgroups (total number of events ≥5 and at least 1 event in both groups), point estimates were below 1.0. In the sensitivity analysis, the crude sHR for the hydrogel spacer group in the entire radiation therapy cohort before IPTW was 0.17 (95% CI, 0.07-0.41, *P* < .001).

**Figure 3:**
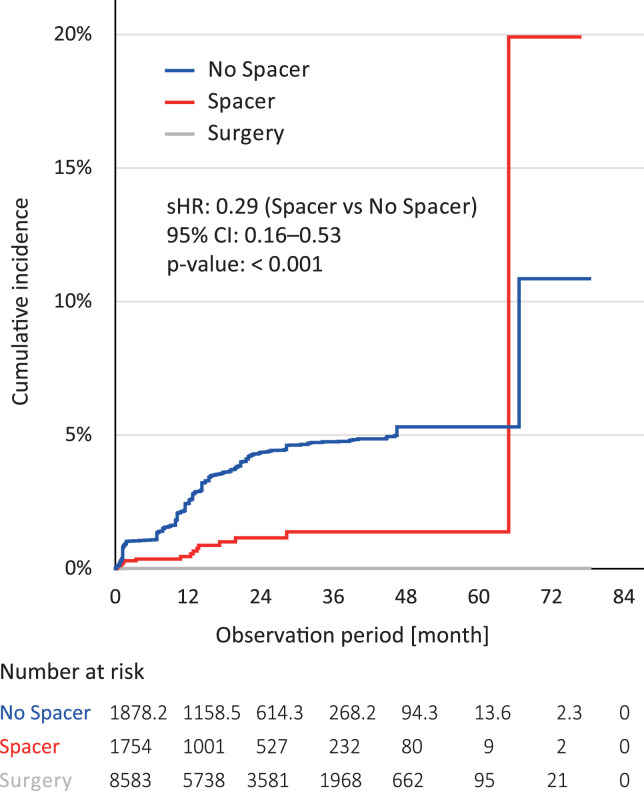
Cumulative incidence of radiation proctitis diagnosis in the propensity score-weighted cohort. The cumulative incidence curves after inverse probability of treatment weighting represent the first diagnosis of radiation proctitis after the initiation of radiation therapy. The subdistribution hazard ratio (sHR) and 95% CI were estimated using a Fine-Gray subdistribution hazard model with death as a competing risk.

**Figure 4:**
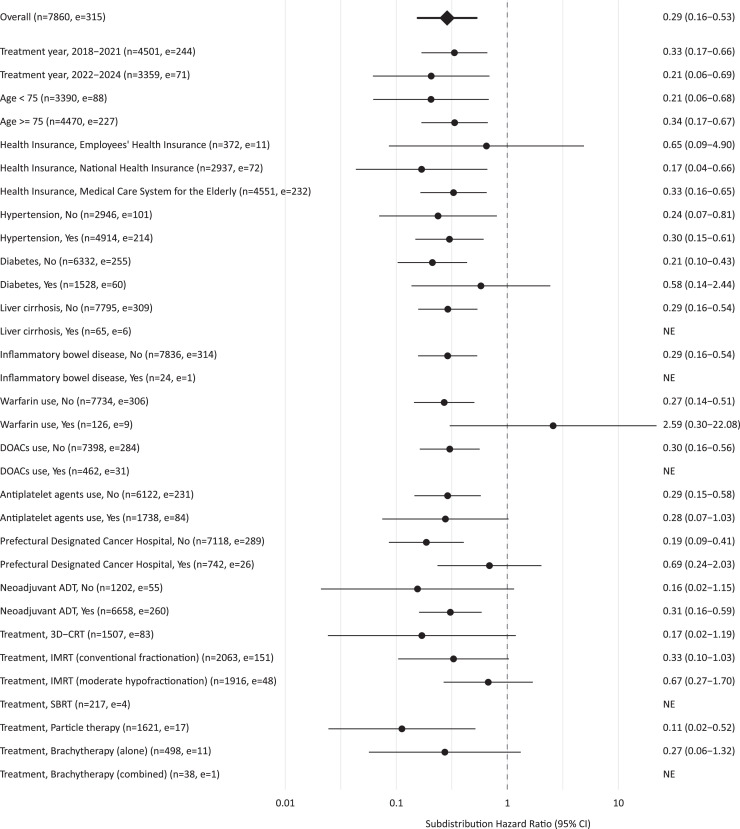
Forest plot of subdistribution hazard ratios for radiation proctitis diagnosis across patient subgroups. The subdistribution hazard ratio and 95% CI for the hydrogel spacer group compared with the nonspacer group in each subgroup are shown. “Not Estimable (NE)” indicates subgroups where the hazard ratio was not calculated due to zero events in one or both arms or a total number of events fewer than 5. *Abbreviations:* 3D-CRT = 3-dimensional conformal radiation therapy; ADT = androgen deprivation therapy; DOACs = direct oral anticoagulants; e = number of events; IMRT = intensity modulated radiation therapy; n = number of patients (before weighting); SBRT = stereotactic body radiation therapy.

For the secondary endpoint, we analyzed the diagnosis of radiation proctitis. The cumulative incidence of events is shown in Fig. 3. The sHR for the hydrogel spacer group was 0.29 (95% CI: 0.16-0.53, *P* < .001). The cumulative incidence of events in patients with and without hydrogel spacer placement was 1.0% versus 3.6% at 18 months, and 1.4% versus 4.8% at 36 months, respectively. For comparison, in the reference surgery group, the cumulative incidence of radiation proctitis diagnosis was 0.01% both at 18 and 36 months. Figure 4 shows a forest plot of sHRs across subgroups. Among the 27 evaluable subgroups, point estimates were below 1.0 in 26 subgroups, except for the warfarin subgroup. In the sensitivity analysis, the crude sHR for the hydrogel spacer group in the entire radiation therapy cohort before IPTW was 0.20 (95% CI, 0.12-0.34, *P* < .001).

## Discussion

This study analyzed 7860 patients from a Japanese real-world claims database and demonstrated that hydrogel spacer placement is significantly associated with a reduction in moderate to severe radiation proctitis requiring invasive procedures. The risk of the primary endpoint, endoscopic gastrointestinal hemostasis, was reduced by approximately 74% (sHR 0.26) in the spacer group compared with the nonspacer group after adjusting for baseline characteristics using propensity score weighting. These findings were consistent across various subgroups, including patients receiving anticoagulants and those treated with particle therapy. To our knowledge, this is one of the largest studies to validate the effectiveness of hydrogel spacer placement in the era of modern radiation therapy practice.

Previous pivotal clinical trials and initial postmarketing data have established that hydrogel spacer placement improves dosimetric parameters and reduces rectal toxicity.^3^^,^^4^^,^^11^^,^^12^ However, these studies were primarily conducted in the era of conventional IMRT and using specific treatment protocols. With the widespread adoption of image guided radiation therapy, moderate to ultrahypofractionation, and particle therapy, overall rectal toxicity has decreased,^5^, ^6^, ^7^, ^8^, ^9^, ^10^ leading to uncertainty regarding the incremental benefit of hydrogel spacer placement in the modern treatment landscape. Recently, Folkert et al addressed this gap using the latest United States claims data, reporting a 46% reduction in rectal procedures associated with spacer use.^15^ Our study corroborates these findings in an independent population, demonstrating that even in an era where rectal doses are optimized, the physical separation provided by spacers confers a substantial protective effect against severe complications requiring endoscopic intervention.

We selected endoscopic gastrointestinal hemostasis as the primary endpoint to serve as a surrogate for clinically significant radiation proctitis requiring invasive procedures, and diagnosis of radiation proctitis as the secondary endpoint for relatively mild radiation proctitis. The 36-month cumulative incidence in the nonspacer group was 2.3% for the primary endpoint and 4.8% for the secondary endpoint. Previous studies have reported National Cancer Institute Common Terminology Criteria for Adverse Events (CTCAE) grade 2 and 3 radiation proctitis in 9% and 1% of patients, respectively, as moderate proctitis,^16^ and grade 1 to 2 toxicity in 5% to 14% as mild proctitis.^16^, ^17^, ^18^ However, the event rates observed in the present study cannot be directly mapped onto these CTCAE-based outcomes. Further validation studies clarifying the correspondence between adverse events identified from claims data and clinical grading are warranted.

A notable finding of this study is the consistent benefit of spacer placement in subgroup analysis, including patients treated with particle therapy. Severe proctitis is reported to be as rare as 1% or lower in modern SBRT or particle therapy^8^^,^^9^^,^^19^ and there is a controversy regarding the clinical effect of spacer placement. However, our data indicate that spacer placement further reduces the risk of bleeding events even in such a subgroup. Interestingly, the utilization rate of spacer placement was higher in the particle therapy and SBRT groups in our unadjusted cohort. This suggests that in clinical practice, physicians may favor spacer use when delivering high fractional doses in SBRT or particle therapy, possibly to mitigate concerns about late toxicity. However, given that rare but serious adverse events associated with spacer placement such as rectal wall necrosis have been reported,^20^, ^21^, ^22^ further research is required that integrates assessments of cost-effectiveness, spacer-related adverse events, and planning effort in treatment planning.

A strength of this study is the application of robust epidemiological methods to the field of radiation oncology using a claims database. Unlike surgery or chemotherapy, radiation therapy billing is often not disease-specific, which has historically limited large-scale claims analysis. By adopting validated definitions and referencing methodologies from other oncological fields, we identified treatment modalities and adverse events. The use of endoscopic hemostasis as an objective outcome minimized the bias often seen with subjective reporting or diagnostic coding, providing a reliable estimate of severe toxicity reduction in a real-world setting. However, there have been few validation studies specifically in the radiation therapy field,^23^ and further standardization of disease definitions and interpretation of billing codes is warranted.

This study has several limitations inherent to its design. First, the algorithms used to identify disease diagnosis and treatment methods, as well as the definitions of adverse event outcomes, have not been validated using clinical records. Consequently, their sensitivity and specificity regarding actual pathological diagnosis or CTCAE grades warrant future verification. Second, the database lacked several clinically relevant factors, including prostate-specific antigen (PSA) levels, stage and risk group, and dose and fractionation. It also lacked information on institutional and physician characteristics that may influence spacer use. These unmeasured variables limited patient characterization and left residual confounding in the IPTW analysis comparing the spacer and nonspacer groups. Third, the follow-up period was relatively short, and a longer observation period is needed for a full assessment of late toxicities. Fourth, the number of cases of ultrahypofractionation, which has become widespread in recent years, was small, limiting the generalizability of our findings to the current treatment landscape. Furthermore, small sample sizes in some subgroups restricted detailed analysis.

## Conclusions

This analysis of a large-scale real-world database demonstrates that hydrogel spacer placement is associated with a significantly lower incidence of radiation proctitis requiring endoscopic hemostasis. These findings support the utility of hydrogel spacer placement in reducing moderate to severe rectal toxicity in the era of modern treatment techniques.

## Disclosures

Shinsaku Okuda reports a relationship with Boston Scientific Corporation that includes: travel reimbursement. Noriyoshi Takahashi reports a relationship with Boston Scientific Corporation that includes: speaking and lecture fees. Keiichi Jingu reports a relationship with Boston Scientific Corporation that includes: speaking and lecture fees and travel reimbursement. Regarding other authors, they declare that they have no known competing financial interests or personal relationships that could have appeared to influence the work reported in this paper.

## Declaration of AI and AI-Assisted Technologies in the Writing Process

During the preparation of this work, the authors used artificial intelligence (AI) services, including ChatGPT, Gemini, Claude, and Cursor, to assist with coding, organizing information, translation, and refining the initial draft. After using these services, the authors reviewed and edited the content as needed and take full responsibility for the content of the publication.
